# Involvement of the Interleukin-23/Interleukin-17 Axis in Chronic Hepatitis C Virus Infection and Its Treatment Responses

**DOI:** 10.3390/ijms17071070

**Published:** 2016-07-15

**Authors:** Ping Meng, Suxian Zhao, Xuemin Niu, Na Fu, Shanshan Su, Rongqi Wang, Yuguo Zhang, Liang Qiao, Yuemin Nan

**Affiliations:** 1 Department of Traditional and Western Medical Hepatology, Third Hospital of Hebei Medical University, Shijiazhuang 050000, China; mengping8547@126.com (P.M.); sxzhao76@163.com (S.Z.); niuxuemin322@163.com (X.N.); funa418@163.com (N.F.); sushanshan1002@126.com (S.S.); wangrongqiw@163.com (R.W.); zygyxa@126.com (Y.Z.); 2 Storr Liver Centre, Westmead Institute for Medical Research (WIMR), the University of Sydney at Westmead Hospital, Westmead, NSW 2145, Australia

**Keywords:** hepatitis C virus, interleukin-23, T helper 17 cells, interferon-γ, myxovirus resistance protein A

## Abstract

Interleukin-23 (IL-23) and its downstream factor IL-17 are the key cytokines involved in immune and inflammatory response in chronic liver diseases. This study aimed to investigate the role and molecular mechanisms of the IL-23/Th17 axis in chronic hepatitis C virus (HCV) infection, and the efficacy of IL-23/Th17 modulation in response to anti-HCV therapy. Sixty-six HCV-infected patients and 20 healthy controls were enrolled. The patients received PegIFNa-2a and ribavirin therapy for at least 48 weeks. The plasma level of IL-23 and the number of IL-17A-, IFN-γ-, and IL-21-producing peripheral blood mononuclear cells (PBMCs) at baseline and 12, 24, and 48 weeks following treatment were determined. The mRNA level of Th17 immune-associated molecules in PBMCs was evaluated by real-time quantitative reverse transcription polymerase chain reaction (qRT-PCR) following treatment with IL-23 agonist or antagonist. Our data showed that, compared to healthy controls, HCV-infected patients had an increased plasma level of IL-23 and increased frequencies of IL-17A- and IFN-γ-producing PBMCs, whereas the HCV patients exhibited a reduced number of IL-21-producing PBMCs. However, the baseline frequencies of IL-21-producing PBMCs were markedly higher in HCV patients who achieved rapid virological response (RVR) than those without RVR. Additionally, the mRNA expressions of IL-21, IFN-γ, myxovirus resistance protein A (MxA), and suppressor of cytokine signaling 3 (SOCS3) were significantly upregulated in PBMCs, while FoxP3 expression was suppressed by IL-23 agonist. Thus, the IL-23/Th17 axis plays an important role in development of chronic HCV infection and antiviral response. IL-23 may enhance the antiviral activity of interferon-based therapy by modulating the expression of Th17 cells-associated molecules in HCV-infected patients.

## 1. Introduction

Hepatitis C virus (HCV) infection remains a critical public health problem, with approximately 185 million people infected worldwide [1]. Recently-developed directing antiviral agents (DAAs) have produced satisfactory therapeutic results in patients with chronic HCV infection. Although DAAs have significantly improved the rate of sustained virological response (SVR), disease progression may not be completely avoidable [2,3]. Pegylated interferon alpha-2a and ribavirin (PegIFNα-2a/RBV) therapy is still the standard treatment for patients with chronic hepatitis C (CHC) in China [4,5]. According to previous studies in China, the SVR rates of full-dose and full-treatment course ranged from 78% to 92.3% [6]. However, the adverse effects and poor tolerance of the combination therapy make it essential to search for more effective treatment strategies for Chinese HCV-infected patients.

Innate and adaptive immunity are closely linked to the prognosis of HCV infection and the response to antiviral therapy [7,8]. In particular, weak cellular immunity against HCV may contribute to the development of chronic infection [9,10]. Although the pathogenesis of the HCV related liver disease is not well understood, the contributing role of T helper 17 (Th17) cells, a newly-described subtype of CD4^+^ T-cells, in chronic hepatitis B (CHB) have been reported [11]. Th17 cells, distinct from other T helper cell subsets including Th1 and Th2, are characterized by the ability to secrete pro-inflammatory cytokines, such as interleukin-17A (IL-17A), IL-21, and IL-22 [12]. IL-23, a member of the IL-12 cytokine family, is necessary for the survival and population expansion of Th17 cells [12] and responsible for inflammatory disorders through its downstream Th17 pathway [13,14]. Studies by Yu et al. [15] have revealed that, in the hepatitis B virus (HBV) patients receiving antiviral therapy with Baraclude, there was a gradual decrease in the serum level of IL-17. Decreased HBV load was associated with decreased frequency of Treg cells, reduced level of IL-23, partially restored T cell immune response, and significant improvement of liver inflammation [16]. However, the role and underlying mechanisms of Th17 cells and related regulating cytokines, especially IL-23 in the development of HCV induced liver diseases, are not yet fully understood.

Thus, the aims of this study were to investigate the role of the IL-23/Th17 axis in chronic HCV infection and its impact on the virological responses in HCV-infected patients undertaking interferon-based therapies.

## 2. Results

### 2.1. Treatment Responses of IFN/RBV Therapy

In a total of 66 patients undertaking antiviral therapy, 39 (59.1%) achieved rapid virological response (RVR). Fifty-seven subjects underwent HCV genotyping; among them 39 patients had genotype 1b infection, of whom 21 patients (53.8%) achieved RVR, 15 patients had genotype 2a infection, of whom 12 (80.0%) patients achieved RVR, and in three cases the genotyping revealed no data. 

### 2.2. Increased Plasma Level of IL-23 in HCV-Infected Patients

**Figure 1:**
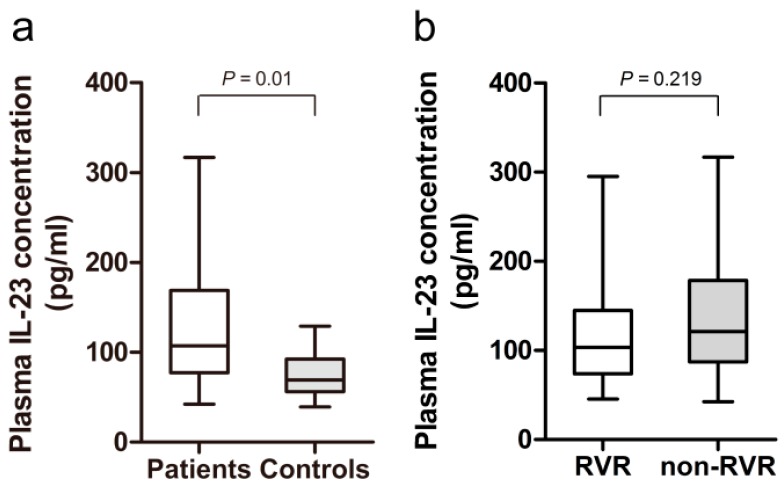
Plasma level of IL-23 was determined by enzyme-linked immunosorbent assay (ELISA). (**a**) Increased plasma level of IL-23 was found in patients with chronic HCV infection (*n* = 48) as compared to the healthy controls (*n* = 10); and (**b**) no significant difference in the baseline plasma concentration of IL-23 was observed between the patients with chronic HCV infection who showed rapid virological response (RVR, *n* = 25) and those with non-RVR (*n* = 23).

HCV infected patients exhibited a significantly higher level of plasma IL-23 than the healthy controls (Figure 1a). No significant difference in the baseline plasma level of IL-23 was observed between the patients with RVR and those with non-RVR (Figure 1b). In the HCV patients, no correlation was found between plasma IL-23 level and baseline viral load (*r* = 0.039, *p* = 0.790).

### 2.3. Associations of IL-17A-, IFN-γ-, and IL-21-Producing Peripheral Blood Mononuclear Cells (PBMCs) with Virological Responses

**Figure 2:**
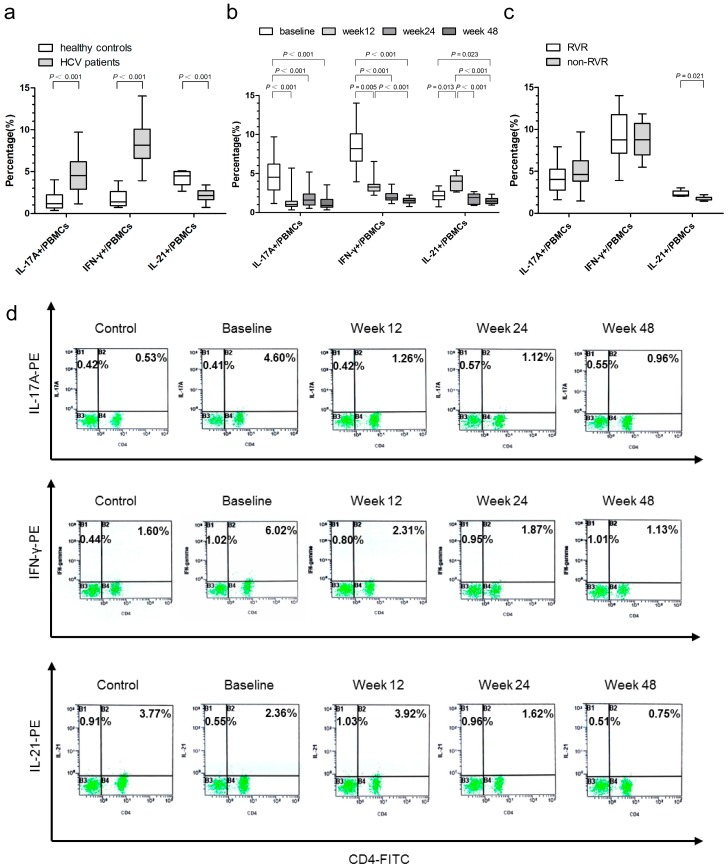
The proportion of IL-17A-, IFN-γ- and IL-21-producing peripheral blood mononuclear cells (PBMCs) were determined by flow cytometry in healthy controls and HCV-infected patients. (**a**) Baseline levels in HCV-infected patients (*n* = 66) and healthy controls (*n* = 20); (**b**) proportion in HCV-infected patients at baseline, 12, 24, and 48 weeks after antiviral therapy; (**c**) comparison of baseline proportion in HCV-infected patients who achieved RVR (*n* = 39) and who had non-RVR (*n* = 27). The length of the box represents the interquartile range. The horizontal line inside each box represents the median values; and (**d**) representative data of IL-17A-, IFN-γ-, and IL-21-producing PBMCs in healthy controls and HCV-infected patients at baseline and 12, 24, and 48 weeks after antiviral therapy. The percentages of CD4^+^/IL-17A^+^, CD4^+^/IFN-γ^+^, and CD4^+^/IL-21^+^ PBMCs are shown on the right upper quadrant of each panel, and the percentages of CD4^−^/IL-17A^+^, CD4^−^/IFN-γ^+^, and CD4^−^/IL-21^+^ PBMCs are shown on the left upper quadrant of each panel.

The proportion of baseline IL-17A- and IFN-γ-producing PBMCs was significantly higher in HCV patients than in healthy controls (Figure 2a). Following the treatment with PegIFNα-2a/RBV for 12 weeks, there was a marked decrease in the percentage of IL-17A- and IFN-γ-producing PBMCs (Figure 2b). Further reduction in the proportion of IFN-γ-producing PBMCs was seen at 24 and 48 weeks of treatment, although the statistical significance was only found between the 48- and 12-week data (Figure 2b). In contrast, the proportion of the baseline IL-21-producing PBMCs was significantly lower in HCV patients than in the healthy controls (Figure 2a), but it increased at 12 weeks of PegIFNα-2a/RBV therapy, decreased to near baseline at 24 weeks, and further reduced at 48 weeks of treatment (Figure 2b). Meanwhile, the proportion of baseline IL-21-producing PBMCs was significantly higher in patients who achieved RVR than in those without RVR (Figure 2c). However, no difference was observed in the proportion of IL-17A- and IFN-γ-producing PBMCs between patients with RVR and those with non-RVR. Representative flow cytometry dot plots of IL-17A-, IFN-γ-, and IL-21-producing PBMCs are presented in Figure 2d.

### 2.4. Impact of IL-23 on the Expression of Th17 Cells Related Immune Molecules

**Figure 3:**
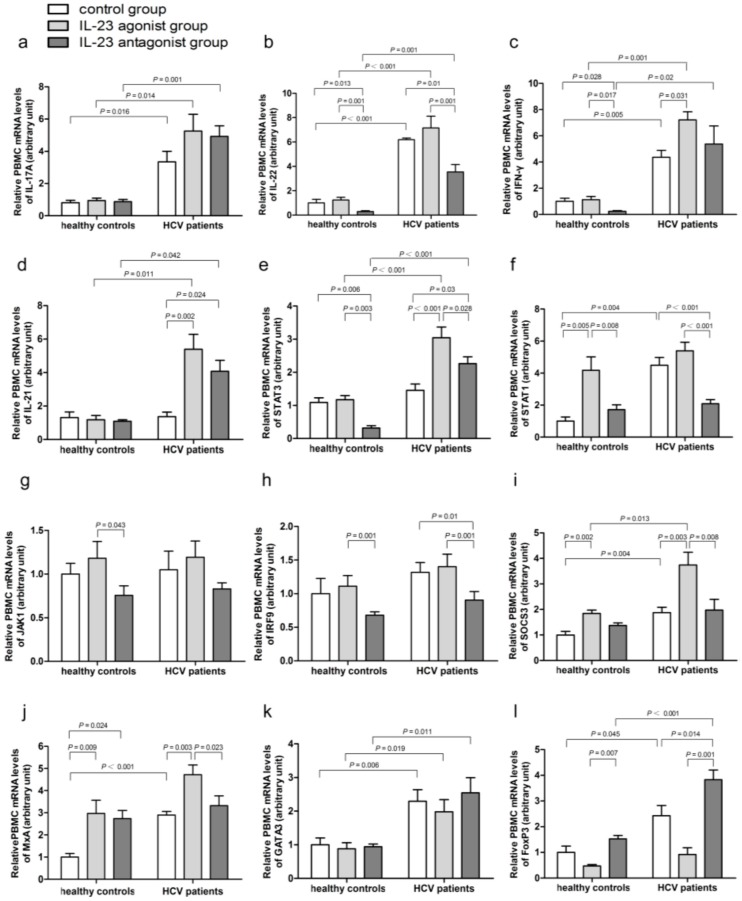
The expressions of IL-17A (**a**); IL-22 (**b**); IFN-γ (**c**); IL-21 (**d**); signal transducer and activator of transcription 3 (STAT3, (**e**)); STAT1 (**f**); Janus kinase 1 (JAK1, (**g**)); interferon regulatory factor 9 (IRF9, (**h**)); suppressor of cytokine signaling 3 (SOCS3, (**i**)); myxovirus resistance protein A (MxA, (**j**)); GATA binding protein 3 (GATA3, (**k**)); and forkhead box P3 (FoxP3, (**l**)) in the PBMCs from healthy controls (*n* = 15) and patients with chronic HCV infection (*n* = 24) were examined by real-time quantitative reverse transcription polymerase chain reaction (qRT-PCR). Data are expressed as mean ± SD.

Compared to the healthy controls, HCV patients of either control group, or IL-23 agonist treatment group or IL-23 antagonist treatment group, showed higher mRNA levels of IL-17A, IL-22, and IFN-γ (Figure 3a–c). Significantly increased mRNA expressions of IL-21 and signal transducer and activator of transcription 3 (STAT3) were observed in HCV patients undertaking IL-23 agonist and antagonist treatment (Figure 3d,e); higher mRNA expressions of STAT1 were only observed in the control group (Figure 3f). There was no significant difference in mRNA expressions of Janus kinase 1 (JAK1) and interferon regulatory factor 9 (IRF9) among the three groups (Figure 3g,h).

In HCV patients, the mRNA expressions of IFN-γ, IL-21, and STAT3 were all elevated in IL-23 agonist group than in the control group (Figure 3c–e). The mRNA expressions of IL-22, STAT3, STAT1, and IRF9 were markedly decreased in the IL-23 antagonist group compared with those in the IL-23 agonist group (Figure 3b,e,f,h). No significant difference was found in mRNA expressions of JAK1 among three groups (Figure 3g).

### 2.5. IL-23 Regulates the mRNA Expressions of SOCS3 and MxA

Higher level of suppressor of cytokine signaling 3 (SOCS3) mRNA expressions were found in the control group and IL-23 agonist group (Figure 3i), while higher mRNA levels of antiviral protein myxovirus resistance protein A (MxA) were only observed in the control group of HCV patients than those of healthy controls (Figure 3j). Furthermore, both SOCS3 and MxA mRNA expressions were increased in IL-23 agonist group and decreased in IL-23 antagonist group of the HCV patients (Figure 3i,j).

### 2.6. IL-23 Alters the mRNA Expressions of the CD4^+^ T-Cells Transcription Factors

Significantly higher mRNA levels of GATA binding protein 3 (GATA3) in PBMCs were found in HCV patients, as compared to healthy controls (Figure 3k). Higher mRNA expression levels of forkhead box P3 (FoxP3) were found in the control group and IL-23 antagonist group of the HCV patients (Figure 3l). No significant difference was found in the mRNA levels of T-box expressed in T cells (T-bet) between the HCV patients and healthy controls (*p* > 0.05).

In healthy controls, FoxP3 mRNA expressions were up-regulated in IL-23 antagonist group compared to those in IL-23 agonist group (Figure 3l); however, in HCV patients, the expression of FoxP3 mRNA was slightly decreased in IL-23 agonist group (Figure 3l) but was inversely upregulated in the IL-23 antagonist group (Figure 3l). No significant difference was observed in the mRNA expressions of GATA3 and T-bet among three groups, both in healthy controls and HCV patients (Figure 3k).

### 2.7. Low Baseline Serum Viral Load May Be a Useful Predictor for Treatment Response in Patients Undertaking PegIFN-α Plus RBV Therapy

We analyzed several variables, including the level of pretreatment IL-21-producing PBMCs, viral load, serum alanine aminotransferase (ALT), and aspartate aminotransferase (AST), to determine if these factors may predict the treatment response. Our data showed that low baseline serum viral load may serve as an important predictor of RVR in HCV patients undergoing dual therapy (OR = 0.525, 95% CI: 0.291–0.947, *p* = 0.032).

### 2.8. Relationship between the Proportion of IL-17A-, IFN-γ-, and IL-21-Producing PBMCs and HCV Genotypes, Baseline Viral Load, ALT, and AST

There was no significant correlation between the proportion of the IL-17A-, IFN-γ-, and IL-21-producing PBMCs and HCV genotypes 1b or 2a in HCV patients, both at the baseline level or following antiviral therapy (*p* > 0.05). Furthermore, no correlation was found between the number of IL-17A-, IFN-γ-, and IL-21-producing PBMCs and the baseline viral load, serum levels of ALT, and AST (*p* > 0.05).

## 3. Discussion

Pathogen-associated molecule patterns (PAMP) can bind to toll-like receptors (TLRs) expressed on antigen-presenting cells (APC, such as dendritic cells and macrophages) to produce IL-23 in HCV infected patients, and the secreted IL-23 could provoke a powerful pro-inflammatory response against HCV by the host immune system [17,18]. In our present study, the baseline plasma levels of IL-23 were markedly higher in HCV-infected patients than in the healthy controls, indicating that IL-23 might be involved in the pathogenesis of hepatic injury in patients with chronic HCV infection. However, the underlying mechanism of IL-23 on regulating chronic HCV infection remains unclear. As we all know, IL-23 could modulate the Th17 cells related molecules to participate in the development and progression of inflammatory disease [13,14]. Therefore, we further explore the role of the IL-23/Th17 axis in chronic HCV infection.

IL-17A is the major cytokine secreted by Th17 cells, and is described as important mediator of inflammation, autoimmunity, and immune defense against certain pathogens [19,20,21,22]. IFN-γ is mainly produced by Th1 cells. Their increasing expressions could well reflect the activation of Th17 and Th1 cells. In our study, the baseline proportion of both IL-17A- and IFN-γ-producing PBMCs were significantly higher in HCV-infected patients than in the healthy controls, indicating that activation of Th17 cells may be involved in development of the HCV induced liver diseases by releasing pro-inflammatory cytokines. Our results are consistent with the data reported by Amaraa et al. [23] and Yasunobu et al. [24], in which the proportion of IFN-γ-producing CD4^+^ T-cells were higher in HCV-infected patients, and the proportion of Th1 cells tended to increase depending on the extent of fibrosis in HCV-infected patients [24,25]. These two studies suggest that Th1 cells might also be involved in HCV induced liver injury. Based on our results and the published data, we propose that activation of lymphocytes, particularly T helper cells expressing IL-17A and IFN-γ, may be pathogenically linked to the progressive hepatic fibrosis in chronic HCV infection. 

In our current study, we also found that the percentage of the IL-17A- and IFN-γ-producing PBMCs were markedly decreased at 12 weeks after PegIFNα-2a plus RBV treatment in HCV patients, and the proportion of the IFN-γ-producing PBMCs further reduced at 24 and 48 weeks, suggesting that PegIFNα-2a plus RBV treatment may alleviate liver injury through suppressing the number of circulating Th1 and Th17 cells. These findings are also consistent with the data reported by Jimenez-Sousa et al. [26], in which the baseline levels of serum IL-17 and IFN-γ in HCV-infected patients could be down-modulated by PegIFNα-2a plus RBV treatment as early as 12 weeks of treatment initiation. Fathy et al. [27] also showed a significant reduction of the serum level of IFN-γ after 12 weeks of treatment with PegIFN plus RBV. All of these data may suggest that the combination therapy may be of more benefit to HCV patients in preventing the immune-driven tissue damage.

As we did not observe any significant difference in the baseline proportion of IL-17A- and IFN-γ-producing PBMCs between the patients with RVR and those with non-RVR, we reasoned that IL-17A and IFN-γ are mainly pro-inflammatory factors that are engaged in HCV damage process, but they may not be reliable markers for the treatment response at an early stage.

IL-21 is mainly produced by activated CD4^+^ T cells and natural killer (NK) T cells [28]. In the present study, HCV patients had lower baseline level of IL-21-producing PBMCs than the healthy controls, but this level significantly increased at 12 weeks of treatment, declined to baseline levels at 24 weeks, and further decreased at 48 weeks. Feng et al. [29] reported that the level of HCV-specific IL-21^+^CD4^+^ T cells was closely related to the disease progression in HCV infection in that the relatively lower proportion of IL-21-producing PBMCs in HCV-infected patients might lead to the persistence of HCV infection. Interferon-based therapy could enhance the adaptive HCV-specific immune response. Thus, antiviral therapy with PegIFNα-2a and RBV could rescue the function of effector T cells [30]. This can be further extrapolated that HCV elimination by the combination anti-viral therapy at an early treatment stage might be achieved by the enhanced number of the IL-21-producing PBMCs. Similarly, in patients with HBV infection, reduction of viral loads following treatment with interferons and nucleoside analogues was associated with increased number of IL-21-producing PBMCs [31]. Moreover, the proportion of baseline IL-21-producing PBMCs were significantly higher in patients with RVR than in those without RVR. We reasoned that a high proportion of baseline IL-21-producing PBMCs is likely a contributing factor for restoring the function of effector T cells in viral infections, as it was reported that the HCV patients with higher baseline serum IL-21 levels were more likely to achieve SVR [32]. In this aspect, IL-21 may be a potential predictor for antiviral treatment in HCV infected individuals. 

In the present study, we also investigated the role of IL-23 on Th17 cells and related immune factors. The baseline mRNA expressions of IL-17A, IFN-γ, and STAT1 in the PBMCs of the HCV patients were significantly higher than those of healthy controls, indicating that these factors may all be involved in the process of HCV-induced inflammatory response and liver damage.

IL-22 was found to be involved in the control of viral infection and prevention of inflammatory injury in many diseases [33]. Our results showed that IL-22 mRNA expressions in all three groups of the HCV patients were markedly higher than those in the healthy controls, suggesting that IL-22 might be an important factor in the alleviation of inflammatory damage in HCV infection and is involved in the maintenance of inflammatory response. Moreover, the mRNA expressions of both IL-21 and STAT3 were markedly increased in HCV-infected patients exposed to IL-23 agonist and IL-23 antagonist. It was reported that production of IL-21 was in a STAT3-dependent manner [34]. Here, the trend of IL-21 expression is consistent with that of STAT3. In PBMCs of HCV-infected patients, the mRNA expressions of IL-21, IFN-γ, and STAT3 were all elevated by IL-23 agonist administration. Previous studies reported that activation of IL-23 signaling by its agonist was involved in maintaining the survival and population expansion of Th17 cells, and IL-21, a product of Th17 cells, could rapidly induce mRNA synthesis for IFN-γ [35]. It is known that STAT3 is engaged in the signal transduction of Th17 cells. Activation of IFN-γ results in receptor oligomerization followed by trans-phosphorylation of JAK1, JAK2 and STAT1. Activation of STAT1 may lead to the recruitment of several co-activators, such as IRF9, thereby activating the downstream transcription factors [36,37]. The mRNA expressions of IL-22, STAT1, STAT3, and IRF9 in PBMCs were all significantly decreased in patients exposed to IL-23 antagonist as compared to those treated with IL-23 agonist, indicating that IL-23 antagonist could counteract the promoter action of IL-23 to Th17 cells.

SOCS proteins function as negative-feedback regulators for cytokine-induced JAK-STAT pathway and could be involved in the pathogenesis of HCV persistence [38]. In our study, increased mRNA expressions of SOCS3 were found in the PBMCs of HCV patients compared to healthy controls, and treatment with IL-23 agonist led to a further increase. Our data are consistent with what were reported in a recent study by Collins et al. [39] in that SOCS3 may be an important factor responsible for the beneficial role of alleviating tissue injury in HCV infection by decreasing the production of pro-inflammatory cytokines. Our data also showed that the SOCS3 mRNA expressions were increased in IL-23 agonist group and decreased in IL-23 antagonist group of HCV patients. Chen et al. [38] found that SOCS3 was a necessary negative regulator of IL-23 signaling by constraining the Th17 cells differentiation. Our studies suggest that activation of JAK-STAT pathway by IL-23 agonist led to an increased SOCS3 mRNA expression which, in turn, is involved in the suppression of inflammatory response and prevention of inflammatory damage. As reported previously, IFN-γ also upregulates SOCS3 expression [40], and this is consistent with our data.

MxA can inhibit viral replication and thus plays a pivotal role in host defense against viral infection [41]. Our studies demonstrated a higher baseline mRNA level of MxA in HCV patients than in the healthy controls, which was in keeping with published studies [42,43]. HCV infection could activate monocytes and subsequently lead to increased production of type I IFN α/β and enhanced MxA secretion. Our data also showed that MxA production could also be stimulated by IL-23 agonist treatment in HCV patients, further suggesting an anti-viral role of IL-23 agonist. 

Three transcription factors, T-bet, GATA3, and FoxP3, are involved in regulating the differentiation of Th1, Th2, and regulatory T cells (Tregs), respectively [44]. We found a higher mRNA level of GATA3 in the PBMCs from HCV patients than in the healthy controls. In contrast, the level of FoxP3 mRNA in the PBMCs of the treatment control and IL-23 antagonist group were higher than that in other groups. Our data are consistent with the previously-reported data in that patients with HCV infection had increased number of Th2 and Tregs, and this was associated with increased levels of GATA3 and FoxP3 expression [45,46]. In our study, treatment of HCV patients with IL-23 antagonist led to an up-regulation of FoxP3 mRNA expressions in PBMCs, as compared to the patients treated with IL-23 agonist or the healthy controls. As Th17-derived IL-6 and IL-21 could block the differentiation of Tregs [47], we propose that increased expressions of IL-21 mRNA by IL-23 agonist could might have caused a corresponding reduction in the FoxP3 mRNA expressions. Importantly, our data on the mRNA expressions of IL-17A, IL-22, STAT1, SOCS3, MxA, and FoxP3 in the PBMCs are consistent with the changes of these cytokines in livers reported in other studies [48,49,50,51,52]. Our data also suggest that the imbalance of T-cell subsets distribution might have important influences on disease progression and clinical outcome.

It should be noted that due to the high complete early virological response (cEVR) rate in the samples we studied, we could not analyze the relationships between serum levels of IL-23 or IL-17A-, IFN-γ- and IL-21-producing PBMCs with rate of cEVR and a larger sample size would be needed for a more accurate analysis.

In summary, our study has revealed that the IL-23/Th17 axis may play an important role in development of chronic HCV infection and antiviral response. IL-23 may enhance the antiviral activity of interferon-based therapy by modulating the expression of Th17 cells-associated molecules in HCV-infected patients.

## 4. Materials and Methods

### 4.1. Subjects

A total of 66 patients with chronic HCV infection were recruited from the Third Hospital of Hebei Medical University from March 2013 to December 2014. Eligible patients were between 18 to 65 years of age. Diagnosis was made using the European Association for the Study of the Liver (EASL) Clinical Practice Guidelines: Management of hepatitis C virus infection [53] and the diagnostic criteria of the “Guideline of Prevention and Treatment for Hepatitis C” by the Chinese Society of Hepatology and Chinese Society of Infectious Diseases and Parasitology of the Chinese Medical Association [54]. Participants with the following conditions were excluded: presence of decompensated cirrhosis; co-infection with human immunodeficiency virus (HIV); co-infection with hepatitis A, B, or D virus; other chronic liver disease or co-morbidities precluding interferon therapy. Twenty age- and sex-matched healthy donors were used as controls. The baseline characteristics of the subjects are shown in Table 1. Peripheral blood samples were collected at baseline (from all healthy controls and the HCV patients) and at weeks 12, 24, and 48 of treatment (from HCV patients). Written informed consent was obtained from all patients. The study protocol was approved by the ethics committees of Third Hospital of Hebei Medical University.

### 4.2. Detection of HCV Antibody, Viral Load, and Genotypes of HCV

Serum HCV antibody was detected by enzyme-linked immunosorbent assay (ELISA) using a commercial detection kit (Livzon Diagnostics Inc., Zhuhai, China). Plasma HCV RNA load was determined using real-time quantitative reverse transcription polymerase chain reaction (qRT-PCR) assay (Cobas Taqman HCV Test, Roche Diagnostics, Indianapolis, IN, USA). With this method, the lowest detection limit is 15 IU/mL. The HCV genotypes were identified using the HCV genotyping oligochip (Tianjin Third Central Hospital, Tianjin, China) [55].

### 4.3. Biochemical Assays

Serum ALT and AST levels were detected by an Olympus AU5400 automatic chemical analyzer (Olympus CO., Ltd., Tokyo, Japan).

### 4.4. Detection of Plasma IL-23

The plasma levels of IL-23 in 10 healthy controls and 48 HCV-infected patients were detected by ELISA (Multi Sciences, Hangzhou, China). The results were analyzed using SOFTmax PRO 4.3 LS software (Molecular Devices, Sunnyvale, CA, USA).

### 4.5. Antiviral Treatments and Assessments of Virological Responses

According to disease status and body weight, enrolled patients were divided into two groups: routine-dose group (*n* = 40, body weight ≥ 60 kg, aged 18–60 years, treated with PegIFNα-2a at 180 μg/week); and low-dose group (*n* = 26, patients with compensated cirrhosis, leukocytopenia, body weight < 60 kg, aged 60–65 years, treated with PegIFNα-2a at 67.5–135 μg/week). As the mean body weights are lower for Chinese than Western patients, the dose of RBV was adjusted as follows: 900 mg/day for patients with body weight < 65 kg, 1000 mg/day if the body weight is 65–85 kg, and 1200 mg/day for those with body weight > 85 kg. In patients with undetectable HCV RNA, the treatment was given for an additional 44 weeks. Standard definitions of virological responses were used to assess the therapeutic effect. RVR was defined as undetectable serum HCV RNA at week 4 of therapy. SVR was defined as undetectable serum HCV RNA levels at 24 weeks post-therapy. PegIFNα-2a was obtained from F. Hoffmann-La Roche Ltd., Basel, Switzerland; and RBV was obtained from Zhejiang Chengyi Pharmaceutical Co., Ltd., Zhejiang, China.

### 4.6. Measurement of IL-17A-, IFN-γ-, and IL-21-Producing PBMCs by Flow Cytometry

FITC-conjugated anti-CD4, PE-conjugated anti-IFN-γ, anti-IL-17A, and anti-IL-21 mAbs were purchased from Beckman Coulter (Marseilles, France), eBioscience (San Diego, CA, USA), and BD Biosciences (San Jose, CA, USA), respectively. For cytokine detection, 500 μL fresh heparinized peripheral blood was incubated in an equal volume RPMI 1640 medium, and stained with anti-CD4, and then activated with 50 ng/mL phorbol 12-myristate 13-acetate (PMA), 1 μg/mL ionomycin (Sigma, St Louis, MO, USA) and 0.7 µL GolgiStop (BD Biosciences) simultaneously for 4 h. After fixation and permeabilization, cells were stained with the above-mentioned specific antibodies. At least 1 × 10^5^ events in the lymphogate were collected by flow cytometry (Beckman Coulter Cytomics FC500) for analysis.

### 4.7. PBMC Isolation and Culture

PBMCs were isolated from fresh blood samples of 24 HCV patients and 15 healthy subjects using Ficoll density gradient centrifugation (Hanyang Biologicals Technology, Tianjin, China). Cell culture was performed in serum free medium (Botna Biological Technology, Beijing, China) supplemented with 100 μg/mL penicillin-streptomycin (North China Pharmaceutical Group Corporation, Hebei, China) and 300 μg/mL l-glutamine (Bio-high Technology, Hebei, China). PBMCs cultured in 24-well plate (1 × 10^6^/well) were divided into three groups: control group (no treatment); IL-23 agonist group (treated with 100 ng/mL human IL-23 recombinant protein); and IL-23 antagonist group (treated with 400 ng/mL anti-human IL-23). All PBMCs were incubated in 5% CO_2_ at 37 °C for 48 h. Both IL-23 recombinant protein and IL-23 antagonist (p19 subtype, purified functional grade) were purchased from eBioscience.

### 4.8. Analysis of mRNA Expression in PBMCs by qRT-PCR

The mRNA levels of IL-17A, IL-21, IL-22, IFN-γ, T-bet, GATA3, FoxP3, JAK1, STAT1, STAT3, IRF9, SOCS3, and MxA in PBMCs were determined by qRT-PCR. Relative mRNA quantification was calculated by 2^−ΔΔ^*^C^*^t^. The specific primer sequences (Sangon Biotech, Shanghai, China) are listed in Table 2.

### 4.9. Statistical Analysis

The statistical power calculation was performed based on predictive significance of RVR to SVR achievement. The target sample size gave 80% power at the 5% significance level to detect a difference of 42.4% in SVR rates (84.9% vs. 42.5%) between the RVR and non-RVR patients according to the outcome of our completed clinical trial. For continuous variables normally distributed, or not normally distributed, values were expressed as mean ± standard deviation (SD), medians, and ranges; Student’s *t*-test or Mann-Whitney *U* test was used to compare the statistical differences between two groups; One-way analysis of variance (ANOVA) or Kruskal-Wallis *H* test was performed for comparisons of values among at least three groups; the least significant difference-*t* (LSD-*t*) test or Nemenyi method was conducted for further comparisons between any two groups. Associations between variables were evaluated using Spearman rank order correlations. Multivariate logistic regression analysis was applied to detect the independent predictors of RVR. Data analysis was performed using SPSS software version 16.0 (SPSS Inc., Chicago, IL, USA) and GraphPad Prism 5.0 software (GraphPad Software Inc., San Diego, CA, USA). A *p* value of <0.05 was considered significant.

## 5. Conclusions

Our study showed that the HCV patients with high baseline levels of IL-21-producing PBMCs are more likely to achieve RVR. Thus, IL-21 could possibly be used as a novel predictor of antiviral therapy with PegIFNα-2a plus RBV. IL-23 can enhance the levels of Th17 cell subsets in PBMCs and further upregulate the expressions of immune factors IL-17A, IL-21, IL-22, IFN-γ, and antiviral protein MxA and suppress the expressions of transcription factors FoxP3 and GATA3, thereby promoting anti-HCV immune response. Thus, IL-23, and its downstream effector Th17 cells, might serve as important immune targets for the treatment of chronic HCV infection.

## Figures and Tables

**Table 1:** Clinical characteristics of the study subjects.

Parameter	HCV Patients (*n* = 66)	Healthy Controls (*n* = 20)	*p*-Value
Gender (M/F)	28/38	8/12	0.774
Age (mean ± SD)	49.2 ± 10.8	45.9 ± 9.37	0.367
BMI (mean ± SD, kg/m^2^)	25.1 ± 3.1	23.8 ± 2.1	0.105
ALT (IU/L), median (range)	48 (15–239)	19 (9–35)	0.000
AST (IU/L), median (range)	39 (15–188)	21 (14–41)	0.000
HCV RNA (median/range, log_10_ IU/mL)	6.26/2.29–7.84	n.d.	
**Possible Route of Contamination**			
Transfusion, *n* (%)	39 (59.09)	--	
Previous surgery, *n* (%)	21 (31.82)	--	
Others or unknown, *n* (%)	6 (9.09)	--	
HCV genotypes (1b/2a)	39/15	--	

ALT: alanine transaminase; AST: aspartate transaminase; BMI: body mass index; M: male; F: female; n.d.: not determined.

**Table 2:** Primers used for qRT-PCR analysis.

Gene	Product Length	Primer Sequences
IL-17A	154 bp	F 5′-AACCGATCCACCTCACCTTG-3′
		R 5′-TCTCTTGCTGGATGGGGACA-3′
IL-21	101 bp	F 5′-CAAATCAAGCTCCCAAGGTC-3′
		R 5′-CAGGGACCAAGTCATTCACA-3′
IL-22	114 bp	F 5′-TATATCACCAACCGCACCTTC-3′
		R 5′-GCGCTCACTCATACTGACTCC-3′
IFN-γ	101 bp	F 5′-TTGGGTTCTCTTGGCTGT-3′
		R 5′-CCATTATCCGCTACATCTGAA-3′
JAK1	101 bp	F 5′-GGATTGCTCCTGAGTGTGTTG-3′
		R 5′-CTCGCCATTGTAGCAGATTTC-3′
STAT1	151 bp	F 5′-CACCCAAAGTATCAGGACGAG-3′
		R 5′-CGTTCCTACGTCAAGCAGTTC-3′
STAT3	109 bp	F 5′-TTTATCAGTAAGGAGCGGGA-3′
		R 5′-CCCAAGTGAAAGTGACGC-3′
IRF9	105 bp	F 5′-CACACGATTGACCTGTCCTCT-3′
		R 5′-TTAGCCTTGAGTTCTCCACCA-3′
MxA	102 bp	F 5′-GCATCCCACCCTCTATTACTG-3′
		R 5′-CACCTTCTCCTCATACTGGCT-3′
T-bet	104 bp	F 5′-TCGTTGGCATGTGTGTTAATC-3′
		R 5′-TGTCCAAAGTCAGGTGAGTCC-3′
GATA3	100 bp	F 5′-AAGCCTAAACGCGATGGATA-3′
		R 5′-AGTGGTTGGAACACAGACACC-3′
FoxP3	122 bp	F 5′-TCCCAGAGTTCCTCCACAAC-3′
		R 5′-ATTGAGTGTCCGCTGCTTCT-3′
SOCS3	114 bp	F 5′-AGGAGACGGGACATCTTTCAC-3′
		R 5′-ATGGGACAGGGAGCATTTAAG-3′

FoxP3: forkhead box P3; GATA3: GATA binding protein 3; IFN-γ: interferon-γ; IL-17A: interleukin-17A; IRF9: interferon regulatory factor 9; JAK1: Janus kinase 1; MxA: myxovirus resistance protein A; SOCS3: suppressor of cytokine signaling 3; STAT1: signal transducer and activator of transcription 1; T-bet: T-box expressed in T cells; F: forward; R: reverse.

## Abbreviations

FoxP3	forkhead box P3
GATA3	GATA binding protein 3
IFN-γ	Interferon-γ
IL-23	Interleukin-23
MxA	myxovirus resistance protein A
PBMC	peripheral blood mononuclear cell
SOCS3	suppressor of cytokine signaling 3
STAT1	signal transducer and activator of transcription 1
T-bet	T-box expressed in T cells

## References

[1] Mohd Hanafiah K., Groeger J., Flaxman A.D., Wiersma S.T. (2013). Global epidemiology of hepatitis c virus infection: New estimates of age-specific antibody to hcv seroprevalence. Hepatology.

[2] Qian X.J., Zhu Y.Z., Zhao P., Qi Z.T. (2016). Entry inhibitors: New advances in hcv treatment. Emerg. Microbes Infect..

[3] Paolucci S., Fiorina L., Mariani B., Landini V., Gulminetti R., Novati S., Maserati R., Barbarini G., Bruno R., Baldanti F. (2015). Development and persistence of DAA resistance associated mutations in patients failing HCV treatment. J. Clin. Virol..

[4] European Association for Study of Liver (2015). EASL recommendations on treatment of hepatitis C 2015. J. Hepatol..

[5] AASLD/IDSA HCV Guidance Panel (2015). Hepatitis C guidance: AASLD-IDSA recommendations for testing, managing, and treating adults infected with hepatitis C virus. Hepatology.

[6] Nan Y.M., Zheng H.W., Sun D.X., An C.M., Li Y.S., Kong L., Dai E.H., Zhang Y.G., Zhao S.X., Su S.S. (2013). Study of using an individualized treatment strategy to treat patients with chronic hepatitis C. Chin. J. Hepatol..

[7] Grakoui A., Shoukry N.H., Woollard D.J., Han J.H., Hanson H.L., Ghrayeb J., Murthy K.K., Rice C.M., Walker C.M. (2003). HCV persistence and immune evasion in the absence of memory T cell help. Science.

[8] Shoukry N.H., Grakoui A., Houghton M., Chien D.Y., Ghrayeb J., Reimann K.A., Walker C.M. (2003). Memory CD8+ T cells are required for protection from persistent hepatitis C virus infection. J. Exp. Med..

[9] Ulsenheimer A., Gerlach J.T., Gruener N.H., Jung M.C., Schirren C.A., Schraut W., Zachoval R., Pape G.R., Diepolder H.M. (2003). Detection of functionally altered hepatitis C virus-specific CD4 T cells in acute and chronic hepatitis C. Hepatology.

[10] Chang K.M. (2003). Immunopathogenesis of hepatitis C virus infection. Clin. Liver Dis..

[11] Zhang J.Y., Zhang Z., Lin F., Zou Z.S., Xu R.N., Jin L., Fu J.L., Shi F., Shi M., Wang H.F. (2010). Interleukin-17-producing CD4(+) T cells increase with severity of liver damage in patients with chronic hepatitis B. Hepatology.

[12] Wilson N.J., Boniface K., Chan J.R., McKenzie B.S., Blumenschein W.M., Mattson J.D., Basham B., Smith K., Chen T., Morel F. (2007). Development, cytokine profile and function of human interleukin 17-producing helper T cells. Nat. Immunol..

[13] Huang Z., van Velkinburgh J.C., Ni B., Wu Y. (2012). Pivotal roles of the interleukin-23/T helper 17 cell axis in hepatitis B. Liver Int..

[14] Wang Q., Zhou J., Zhang B., Tian Z., Tang J., Zheng Y., Huang Z., Tian Y., Jia Z., Tang Y. (2013). Hepatitis B virus induces IL-23 production in antigen presenting cells and causes liver damage via the IL-23/IL-17 axis. PLoS Pathog..

[15] Yu X.H., Zhang J.C., Li X.Y., Chen T. (2015). Changes to the migratory inhibitory factor, IL-17, and IL-10 levels in serum from chronic hepatitis B patients and clinical significance following baraclude^®^ treatment. Genet. Mol. Res..

[16] Su Z.J., Yu X.P., Guo R.Y., Ming D.S., Huang L.Y., Su M.L., Deng Y., Lin Z.Z. (2013). Changes in the balance between treg and Th17 cells in patients with chronic hepatitis B. Diagn. Microbiol. Infect. Dis..

[17] Ashrafi Hafez A., Ahmadi Vasmehjani A., Baharlou R., Mousavi Nasab S.D., Davami M.H., Najafi A., Joharinia N., Rezanezhad H., Ahmadi N.A., Imanzad M. (2014). Analytical assessment of interleukin-23 and -27 cytokines in healthy people and patients with hepatitis C virus infection (genotypes 1 and 3a). Hepat. Mon..

[18] Wang J.M., Shi L., Ma C.J., Ji X.J., Ying R.S., Wu X.Y., Wang K.S., Li G., Moorman J.P., Yao Z.Q. (2013). Differential regulation of interleukin-12 (IL-12)/IL-23 by tim-3 drives T(h)17 cell development during hepatitis C virus infection. J. Virol..

[19] Zhao L., Tang Y., You Z., Wang Q., Liang S., Han X., Qiu D., Wei J., Liu Y., Shen L. (2011). Interleukin-17 contributes to the pathogenesis of autoimmune hepatitis through inducing hepatic interleukin-6 expression. PLoS ONE.

[20] Happel K.I., Dubin P.J., Zheng M., Ghilardi N., Lockhart C., Quinton L.J., Odden A.R., Shellito J.E., Bagby G.J., Nelson S. (2005). Divergent roles of IL-23 and IL-12 in host defense against klebsiella pneumoniae. J. Exp. Med..

[21] Infante-Duarte C., Horton H.F., Byrne M.C., Kamradt T. (2000). Microbial lipopeptides induce the production of IL-17 in Th cells. J. Immunol..

[22] Huang W., Na L., Fidel P.L., Schwarzenberger P. (2004). Requirement of interleukin-17A for systemic anti-candida albicans host defense in mice. J. Infect. Dis..

[23] Amaraa R., Mareckova H., Urbanek P., Fucikova T. (2002). T helper, cytotoxic T lymphocyte, NK cell and NK-T cell subpopulations in patients with chronic hepatitis C. Folia Microbiol..

[24] Kawakami Y., Nabeshima S., Furusyo N., Sawayama Y., Hayashi J., Kashiwagi S. (2000). Increased frequency of interferon-gamma-producing peripheral blood CD4+ T cells in chronic hepatitis C virus infection. Am. J. Gastroenterol..

[25] Matsui T., Nagai H., Sumino Y., Miki K. (2008). Relationship of peripheral blood CD4-positive T cells to carcinogenesis in patients with HCV-related chronic hepatitis and liver cirrhosis. Cancer Chemother. Pharmacol..

[26] Jimenez-Sousa M.A., Almansa R., de la Fuente C., Caro-Paton A., Ruiz L., Sanchez-Antolin G., Gonzalez J.M., Aller R., Alcaide N., Largo P. (2010). Increased Th1, Th17 and pro-fibrotic responses in hepatitis C-infected patients are down-regulated after 12 weeks of treatment with pegylated interferon plus ribavirin. Eur. Cytokine Netw..

[27] Fathy A., Ahmed A.S., Metwally L., Hassan A. (2011). T helper type 1/T helper type 17-related cytokines in chronic hepatitis C patients before and after interferon and ribavirin therapy. Med. Princ. Pract..

[28] Parrish-Novak J., Dillon S.R., Nelson A., Hammond A., Sprecher C., Gross J.A., Johnston J., Madden K., Xu W., West J. (2000). Interleukin 21 and its receptor are involved in NK cell expansion and regulation of lymphocyte function. Nature.

[29] Feng G., Zhang J.Y., Zeng Q.L., Jin L., Fu J., Yang B., Sun Y., Jiang T., Xu X., Zhang Z. (2013). HCV-specific interleukin-21+CD4+ T cells responses associated with viral control through the modulation of HCV-specific CD8+ T cells function in chronic hepatitis C patients. Mol. Cells.

[30] Feld J.J., Hoofnagle J.H. (2005). Mechanism of action of interferon and ribavirin in treatment of hepatitis C. Nature.

[31] Ma S.W., Huang X., Li Y.Y., Tang L.B., Sun X.F., Jiang X.T., Zhang Y.X., Sun J., Liu Z.H., Abbott W.G. (2012). High serum IL-21 levels after 12 weeks of antiviral therapy predict HBeAg seroconversion in chronic hepatitis B. J. Hepatol..

[32] Hsu C.S., Hsu S.J., Liu W.L., Chen C.L., Liu C.J., Chen P.J., Chen D.S., Kao J.H. (2013). IL-21R gene polymorphisms and serum IL-21 levels predict virological response to interferon-based therapy in asian chronic hepatitis C patients. Antivir. Ther..

[33] Witte E., Witte K., Warszawska K., Sabat R., Wolk K. (2010). Interleukin-22: A cytokine produced by T, NK and NKT cell subsets, with importance in the innate immune defense and tissue protection. Cytokine Growth Factor Rev..

[34] Wei L., Laurence A., Elias K.M., O’Shea J.J. (2007). IL-21 is produced by Th17 cells and drives IL-17 production in a STAT3-dependent manner. J. Biol. Chem..

[35] Strengell M., Sareneva T., Foster D., Julkunen I., Matikainen S. (2002). IL-21 up-regulates the expression of genes associated with innate immunity and Th1 response. J. Immunol..

[36] Saha B., Jyothi Prasanna S., Chandrasekar B., Nandi D. (2010). Gene modulation and immunoregulatory roles of interferon gamma. Cytokine.

[37] Schroder K., Hertzog P.J., Ravasi T., Hume D.A. (2004). Interferon-γ: An overview of signals, mechanisms and functions. J. Leukoc. Biol..

[38] Chen Z., Laurence A., Kanno Y., Pacher-Zavisin M., Zhu B.M., Tato C., Yoshimura A., Hennighausen L., O’Shea J.J. (2006). Selective regulatory function of SOCS3 in the formation of IL-17-secreting T cells. Proc. Natl. Acad. Sci. USA.

[39] Collins A.S., Ahmed S., Napoletano S., Schroeder M., Johnston J.A., Hegarty J.E., O’Farrelly C., Stevenson N.J. (2014). Hepatitis C virus (HCV)-induced suppressor of cytokine signaling (Socs) 3 regulates proinflammatory TNF-alpha responses. J. Leukoc. Biol..

[40] Gatto L., Berlato C., Poli V., Tininini S., Kinjyo I., Yoshimura A., Cassatella M.A., Bazzoni F. (2004). Analysis of Socs-3 promoter responses to interferon gamma. J. Biol. Chem..

[41] Staeheli P., Pitossi F., Pavlovic J. (1993). Mx proteins: Gtpases with antiviral activity. Trends Cell Biol..

[42] Shaker O., Ahmed A., Doss W., Abdel-Hamid M. (2010). MxA expression as marker for assessing the therapeutic response in HCV genotype 4 Egyptian patients. J. Viral Hepat..

[43] Meier V., Mihm S., Ramadori G. (2000). MxA gene expression in peripheral blood mononuclear cells from patients infected chronically with hepatitis C virus treated with interferon-alpha. J. Med. Virol..

[44] Oestreich K.J., Weinmann A.S. (2012). Encoding stability versus flexibility: Lessons learned from examining epigenetics in T helper cell differentiation. Curr. Top. Microbiol. Immunol..

[45] Hao C., Zhou Y., He Y., Fan C., Sun L., Wei X., Wang L., Peng M., Wang P., Lian J. (2014). Imbalance of regulatory T cells and T helper type 17 cells in patients with chronic hepatitis C. Immunology.

[46] Trapero-Marugan M., Garcia-Buey L., Munoz C., Quintana N.E., Moreno-Monteagudo J.A., Borque M.J., Fernandez M.J., Salvanes F.R., Medina J., Moreno-Otero R. (2006). Sustained virological response to peginterferon plus ribavirin in chronic hepatitis C genotype 1 patients is associated with a persistent Th1 immune response. Aliment. Pharmacol. Ther..

[47] Monteleone G., Pallone F., MacDonald T.T. (2008). Interleukin-21: A critical regulator of the balance between effector and regulatory T-cell responses. Trends Immunol..

[48] Chang Q., Wang Y.K., Zhao Q., Wang C.Z., Hu Y.Z., Wu B.Y. (2012). Th17 cells are increased with severity of liver inflammation in patients with chronic hepatitis C. J. Gastroenterol. Hepatol..

[49] El-Saadany S., Ziada D.H., El Bassat H., Farrag W., El-Serogy H., Eid M., Abdallah M., Ghazy M., Salem H.A. (2013). The role of hepatic expression of stat1, SOCS3 and Pias1 in the response of chronic hepatitis C patients to therapy. Can. J. Gastroenterol..

[50] Mihm S., Frese M., Meier V., Wietzke-Braun P., Scharf J.G., Bartenschlager R., Ramadori G. (2004). Interferon type I gene expression in chronic hepatitis C. Lab. Investig..

[51] Wu L.Y., Liu S., Liu Y., Guo C., Li H., Li W., Jin X., Zhang K., Zhao P., Wei L. (2015). Up-regulation of interleukin-22 mediates liver fibrosis via activating hepatic stellate cells in patients with hepatitis C. Clin. Immunol..

[52] Amoras Eda S., Gomes S.T., Freitas F.B., Santana B.B., Ishak G., Ferreira de Araujo M.T., Demachki S., Conde S.R., Ishak Mde O., Ishak R. (2016). Intrahepatic mRNA expression of FAS, FASL, and FOXP3 genes is associated with the pathophysiology of chronic HCV infection. PLoS ONE.

[53] European Association for Study of Liver (2014). EASL clinical practice guidelines: Management of hepatitis C virus infection. J. Hepatol..

[54] Wei L., Hou J.L. (2015). The guideline of prevention and treatment for hepatitis C: A 2015 Update. Zhonghua Gan Zang Bing Za Zhi.

[55] Sun Z.H., Yang H.L., Wei M., Wang S.Y., Wang C.R., Shi Y.L., Ma W.L. (2007). Preparation and application of oligo microarrays for hepatitis virus detection and genotyping. Zhonghua Gan Zang Bing Za Zhi.

