# Correction: Ping Meng, et al. Involvement of the Interleukin-23/Interleukin-17 Axis in Chronic Hepatitis C Virus Infection and Its Treatment Responses. *Int. J. Mol. Sci.* 2016, *17*, 1070

**DOI:** 10.3390/ijms17111793

**Published:** 2016-10-26

**Authors:** Ping Meng, Suxian Zhao, Xuemin Niu, Na Fu, Shanshan Su, Rongqi Wang, Yuguo Zhang, Yuemin Nan, Liang Qiao

**Affiliations:** 1 Department of Traditional and Western Medical Hepatology, Third Hospital of Hebei Medical University, Shijiazhuang 050000, China; mengping8547@126.com (P.M.); sxzhao76@163.com (S.Z.); niuxuemin322@163.com (X.N.); funa418@163.com (N.F.); sushanshan1002@126.com (S.S.); wangrongqiw@163.com (R.W.); zygyxa@126.com (Y.Z.); 2 Storr Liver Centre, Westmead Institute for Medical Research (WIMR), the University of Sydney at Westmead Hospital, Westmead, NSW 2145, Australia

The authors wish to make a change to their published paper [1]. The correct form of “Correspondence” should be: “Correspondence: yueminnan@163.com (Y.N.); Tel.: +86-311-6678-1227 (Y.N.); Fax: +86-311-6678-1289 (Y.N.); qlia8530@usyd.edu.au (L.Q.); Tel.: +612-8627-3534 (L.Q.); Fax: +612-8627-3099 (L.Q.).” The authors apologize for any inconvenience the change may cause.

The change does not affect the scientific results. The manuscript will be updated and the original will remain online on the article webpage.
